# Bilateral symmetrical deep gray matter involvement and leptomeningeal enhancement in a child with MOG-IgG-associated encephalomyelitis

**DOI:** 10.1186/s12883-020-02041-3

**Published:** 2021-01-08

**Authors:** Weibing Shen, Yaner Zhang, Chenguang Zhou, Yaoyao Shen

**Affiliations:** 1 Department of Neurology, The Jinjiang Anhai Hsopital, Jinjiang, Fujian Province China; 2 Department of Neurology, The Fifth Affiliated Hospital of Zhengzhou University, Zhengzhou, Henan Province China; 3 Department of Neurology, Jiangxi Provincial People’s Hospital Affiliated to Nanchang University, No.92 Aiguo Road, Donghu District, Nanchang, 330006 Jiangxi Province China

**Keywords:** Leptomeningeal enhancement, Myelin oligodendrocyte glycoprotein, Deep gray matter, Aseptic meningitis

## Abstract

**Background:**

Currently, myelin oligodendrocyte glycoprotein (MOG)-IgG-associated encephalomyelitis (MOG-EM) is regarded as an independent inflammatory demyelinating disease. Magnetic resonance imaging (MRI) abnormalities occur in 44.4% of patients with MOG-EM. However, symmetrical deep gray matter involvement with leptomeningeal enhancement is rarely described in the literature.

**Case presentation:**

A 3-year-old boy was admitted to our hospital because of acute onset fever, headache, vomiting and disturbance of consciousness. Neurological examination showed somnolence, neck stiffness and positive Kernig’s sign. Brain MRI demonstrated bilateral symmetrical lesions in the basal ganglia and thalamus as well as diffuse leptomeningeal enhancement along the sulci of bilateral hemisphere. Cerebrospinal fluid analysis demonstrated increased cell count (7 cells/mm3, mononuclear cells dominant) and protein (1.17 g/L) without glucose and chloride abnormality. Work-up for infectious and autoimmune causes, serum MOG IgG was positive by cell based assay. Therefore, a diagnosis of MOG-EM was established according to the international recommendatory criteria in 2018. He was administrated with intravenous methylprednisolone followed by oral corticosteroids and had recovered completely within 1 week.

**Conclusions:**

In the setting of meningoencephalitis-like clinical presentation with bilateral symmetrical deep gray matter involvement, MOG-EM should be distinguished from other infectious and autoimmune disorders, such as Epstein-Barr virus (EBV) encephalitis, Japanese encephalitis and Anti-NMDA receptor (NMDAR) encephalitis. Besides, aseptic meningitis associated with leptomeningeal enhancement may be an atypical phenotype of MOG-EM.

## Background

Myelin oligodendrocyte glycoprotein (MOG) is a glycoprotein localized on the outer surface of the myelin sheath and oligodendrocytes. The MOG antibody has been identified in inflammatory demyelinating diseases (IDDs), including acute disseminated encephalomyelitis (ADEM), neuromyelitis optica spectrum disorders (NMOSDs), optic neuritis (ON), transverse myelitis (TM), clinically isolated syndrome, and multiple sclerosis (MS). Nowadays, most experts regard MOG-IgG-associated encephalomyelitis (MOG-EM) as an independent entity, immunopathogenetically distinct from other IDDs. It is estimated that the incidence of magnetic resonance imaging (MRI) abnormalities in patients with MOG-EM is 44.4% [1].. However, bilateral symmetrical deep gray matter involvement with leptomeningeal enhancement is rarely described in the literature.

## Case presentation

**Fig. 1:**
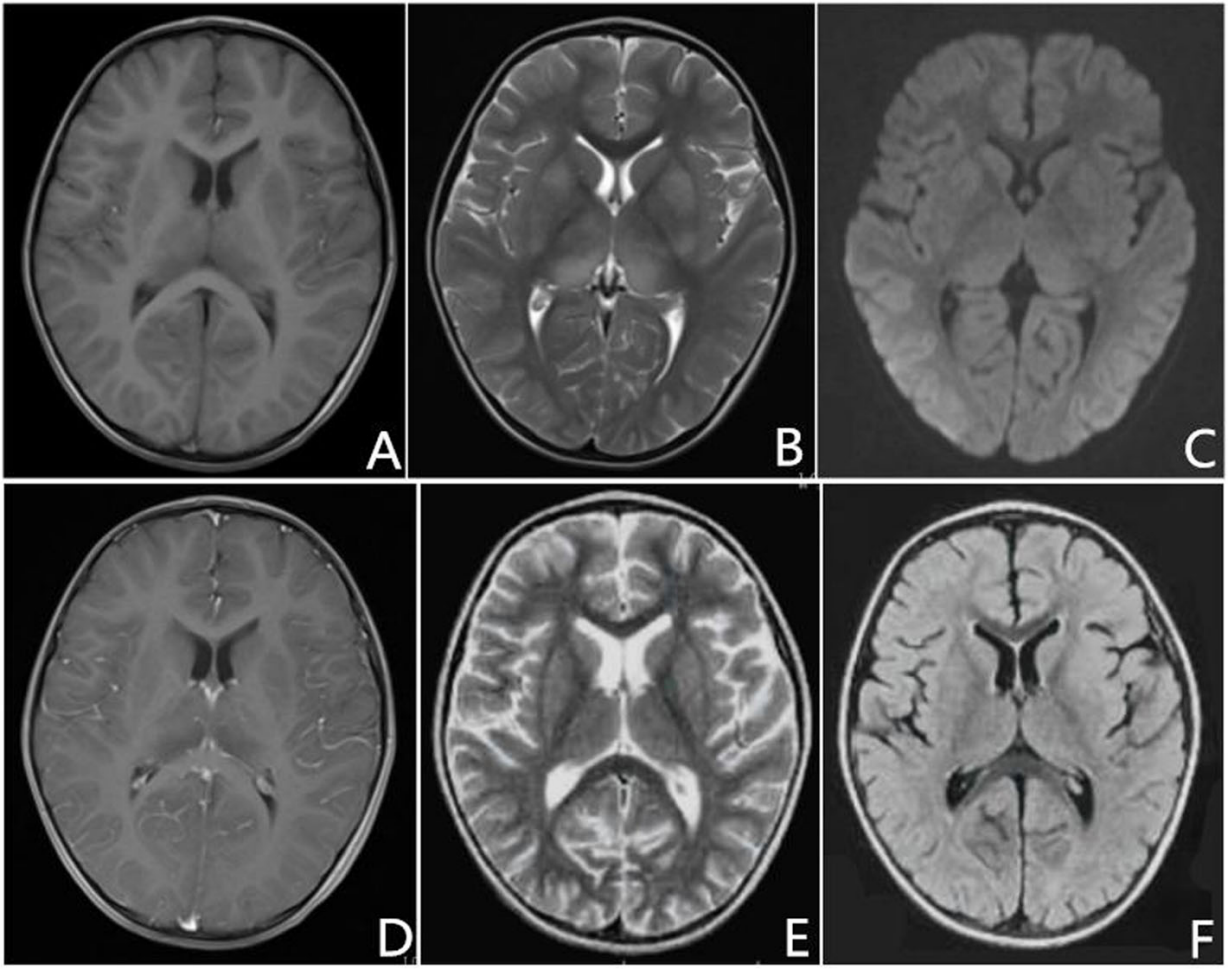
**a**, **b** On day 2 after admission, brain MRI showed bilateral lenticular nucleus and thalamus lesions, hypointense on T1-weighted image and hyperintense on T2-weighted image. **c** None of the lesions showed diffusion restriction. **d** Post contrast T1 axial image showed bilateral leptomeningeal enhancement. **e**, **f** On day 17 after admission, a follow-up MRI examination demonstrated no residual lesions on T2-weighted and FLAIR sequences

A 3-year-old boy with a normal antenatal and developmental history presented with acute onset fever, headache, and vomiting for 5 days, followed by reduced level of consciousness for 2 days prior to admission. On admission, physical examination showed elevated body temperature (37.5 °C) and slightly increased heart rate (104 beats/min) with normal rhythm. Neurological examination revealed somnolence, neck stiffness and positive Kernig’s sign without any other focal neurological deficit. Deep tendon reflexes were present and symmetrical. There was no change in bladder and bowel habits. A increase of leukocyte cell counts (14.000 cells/μL with 78% neutrophils), erythrocyte sedimentation rate (30 mm/h) and C-reactive protein (17 mg/l) was noted on routine laboratory investigations. On day 2 after admission, brain MRI demonstrated bilateral symmetrical lesions in the basal ganglia and thalamus, hyperintense on T2-weighted image and hypointense on T1-weighted image without restricted diffusion (Fig. 1a-c). Furthermore, diffuse leptomeningeal enhancement was noted along the sulci of bilateral hemisphere on the gadolinium-enhanced T1-weighted MRI (Fig. 1d). Bilateral optic nerves and spinal cord were normal. Cerebrospinal fluid analysis demonstrated increased cell count (7 cells/mm3, mononuclear cells dominant) and protein (1.17 g/L) without glucose and chloride abnormality. An electroencephalogram showed diffuse slow waves. Our patient was initially administrated with acyclovir and ceftriaxone for suspected infectious encephalitis. However, symptom of drowsiness was still persistent. Comprehensive work-up for infectious pathogens, including smears and cultures (tuberculosis, other bacteria, and fungi), viral polymerase chain reaction (herpes simplex virus I, II, Epstein-Barr virus, cytomegalovirus, rubella virus), and antibodies against *Borrelia burgdorferi,* were negative in CSF and serum. Moreover, he was routinely tested for human immunodeficiency virus (HIV) and syphilis and found to be negative for both. Autoimmune encephalitis-related autoantibodies, including N-methyl-D-aspartate-receptor (NMDAR) antibodies, contactin associated protein 2 (CASPR2) antibodies, leucine-rich glioma inactivated 1 (LGI1) antibodies, α-amino-3-hydroxy-5-methyl-isoxazolepropionic acid receptor (AMPAR) antibodies, and gamma-aminobutyric acid (GABA) receptor antibodies, were negative both in serum and CSF. On day 6 after admission, established cell-based immunoassays revealed negative serum anti-AQP4 antibodies but positive anti-MOG antibodies with a titer of 1:100. The patient completed 3 days of intravenous methylprednisolone (30 mg/kg) followed by oral prednisolone (2 mg/kg). He had recovered completely within 1 week after initiation of steroid treatment. On day 17 after admission, A follow-up MRI examination demonstrated no residual lesions (Fig. 1e, f).

## Discussion and conclusion

In children, bilateral symmetrical signal intensity alterations in the basal ganglia and thalamus can be caused by a wide spectrum of inherited or acquired neurological disorders. Based on acute onset of fever and headache, disturbance of consciousness, positive meningeal irritation sign with elevated cell count and protein, a diagnosis of meningoencephalitis could be initially established. Hence, infectious and autoimmune causes should be preferentially taken into consideration. Subsequently, positive anti-MOG antibodies was detected in serum and the patient received good clinical outcome after modified corticosteroid therapy. Therefore, MOG-EM could be diagnosed according to the international recommendatory criteria proposed by Jarius S et al., [2].

Supratentorial MRI abnormalities presented at onset is estimated to be 35.4% in MOG-EM patients [3]. Lesions are mostly bilateral, often involving the deep white matter, and brain-stem is easily involved structure at onset of disease. In addition, the deep gray matter can also been frequently affected. Mao L et al., retrospective reviewed 50 Chinese cases with MOG-EM, and the 29% of whom had the deep gray matter involvement, including thalamus and basal ganglia [4]. However, the symmetrical basal ganglia and thalamus involvement is rarely reported. Symptoms of supratentorial brain lesions include headache, fatigue, psychomotor slowing, disorientation, impaired consciousness, hemihypesthesia, meningism, photophobia, and dyskinesia. It’s worth noting that leptomeningeal enhancement can be found in contrast MRI. To our knowledge, leptomeningeal enhancement has been described in MS and NMOSD, previously. Whereas, aseptic meningitis associated with radiological evidence of leptomeningeal enhancement has been relatively rare reported in MOG-EM [5]. Previously, aseptic meningitis can be induced in CD28-deficient C57BL/6 mice after immunization with MOG in animal studies, which indicates that aseptic meningitis may be a direct manifestation of MOG-Ab related disease [6].

In the setting of meningoencephalitis-like clinical presentation with bilateral symmetrical deep gray matter involvement, MOG-EM should be distinguished from other infectious and autoimmune disorders, such as Epstein-Barr Virus (EBV) encephalitis, Japanese encephalitis and anti-NMDA receptor (NMDAR) encephalitis [7]. Of all the members of the *Herpesviridae* family, EBV shows a propensity to affect the deep gray nuclei. T2 hyperintensity in the bilateral basal ganglia and thalami is the typical MRI feature of EBV encephalitis. Other associated lesions include the cerebral hemispheres, brainstem, corpus callosum, and cerebellum. Japanese encephalitis is a mosquito-borne flaviviral encephalitis. Japanese encephalitis virus (JEV) transmission occurs round the year whereas seasonal epidemics begin during the rainy seasons when the mosquito density is maximum. Likewise, bilateral symmetrical deep gray nuclei involvement is the characteristic imaging finding in Japanese encephalitis. Although the child live in South China, there is insufficient evidence for the diagnosis of Japanese encephalitis due to a lack of history of mosquito bites and the onset time of illness being out of epidemic seasons. Moreover, Japanese encephalitis has a poorer prognosis than MOG-EM. Twenty to 40 % of patients with Japanese encephalitis die during the acute stage and about 50% of the survivors have severe neurological sequelae [8]. Anti-NMDAR encephalitis is the most common type of autoimmune encephalitis, which is associated with autoantibodies against neurosurface or synaptic antigens. Bilateral symmetrical basal ganglia necrosis has been described in a case of anti-NMDAR encephalitis presenting with chronic progressive dystonia [9]. Moreover, symmetrical lesions in the bilateral thalami and basal ganglia has also been report in JEV-induced anti-NMDAR encephalitis [10]. When clinical presentation is characterized by aseptic meningitis, a comprehensive work-up for infectious and autoimmune etiologies are critical to enable accurate diagnosis.

In conclusion, aseptic meningitis associated with leptomeningeal enhancement may be an atypical phenotype of MOG-EM. MOG-EM should be considered for the etiological spectrum of bilateral symmetrical deep gray matter involvement.

## Data Availability

All data related to this case report are documented within this manuscript.

## Abbreviations

MRI: Magnetic resonance imaging
MOG: Myelin oligodendrocyte glycoprotein
IDDs: Inflammatory demyelinating diseaseas
ADEM: Acute disseminated encephalomyelitis
MS: Multiple sclerosis
NMOSDs: Neuromyelitis optica spectrum disorders
ON: Optic neuritis
TM: Transverse myelitis
EBV: Epstein-Barr virus
HIV: Human immunodeficiency virus
NMDAR: N-methyl-D-aspartate-receptor
CASPR2: Contactin associated protein 2
LGI1: Leucine-rich glioma inactivated 1
AMPAR: α-amino-3-hydroxy-5-methyl-isoxazolepropionic acid receptor
GABA: Gamma-aminobutyric acid
